# Supplementary material for: Inferring Time-Lagged Causality Using the Derivative of Single-Cell Expression
Source: Int J Mol Sci. 2022 Mar 20;23(6):3348. doi: 10.3390/ijms23063348 (PMC8948830; doi:10.3390/ijms23063348)
Supplement: Supplementary file 1 [file ijms-23-03348-s001.zip › ijms-1515528-supplementary.pdf]

Edges	Approach 1	Approach 2	Approach 3/ CITL	
Precision	0.751 (0.234)	0.945 (0.106)	0.944 (0.057)	
Recall	0.100 (0.131)	0.154 (0.076)	0.463 (0.102)	
F-measure	0.262 (0.163)	0.259 (0.109)	0.614 (0.094)	
**Directions**	**Approach 1**	**Approach 2**	**Approach 3**	**CITL**
Precision	0.072 (0.101)	0.776 (0.210)	0.359 (0.229)	0.86 5(0.095)
Recall	0.016 (0.043)	0.124 (0.067)	0.172 (0.112)	0.425 (0.105)
F-measure	0.084 (0.075)	0.208 (0.097)	0.234 (0.144)	0.564 (0.104)

Table S1 Comparisons of different approaches based on PC-stable in multi-trace simulations. The average values from 500 multi-trace simulations are shown with standard deviation values in brackets. The significantly best (t-test) results are marked in red.

Edges	Approach 1	Approach 2	Approach 3/CITL	
Precision	0.970 (0.029)	0.982 (0.065)	0.956 (0.056)	
Recall	0.688 (0.102)	0.146 (0.096)	0.392 (0.089)	
F-measure	0.800 (0.073)	0.247 (0.135)	0.549 (0.088)	
**Directions**	**Approach 1**	**Approach 2**	**Approach 3**	**CITL**
Precision	0.403 (0.125)	0.180 (0.179)	0.114 (0.090)	0.637 (0.162)
Recall	0.291 (0.110)	0.028 (0.031)	0.049 (0.043)	0.258 (0.080)
F-measure	0.336 (0.116)	0.069 (0.044)	0.082 (0.052)	0.363 (0.100)

Table S2 Comparisons of different approaches based on PC-stable for instant causalities in single-trace simulations. The averages of 500 single-trace simulations are shown with standard deviation values in brackets. The significantly best (t-test) results are marked in red.

Edges	Approach 1	Approach 2	Approach 3/CITL	
Precision	0.971 (0.029)	0.982 (0.050)	0.953 (0.059)	
Recall	0.689 (0.105)	0.144 (0.095)	0.394 (0.089)	
F-measure	0.802 (0.075)	0.246 (0.133)	0.551 (0.089)	
**Directions**	**Approach 1**	**Approach 2**	**Approach 3**	**CITL**
Precision	0.406 (0.130)	0.175 (0.178)	0.120 (0.099)	0.633 (0.164)
Recall	0.294 (0.115)	0.027 (0.029)	0.053 (0.049)	0.258 (0.080)
F-measure	0.340 (0.120)	0.067 (0.041)	0.089 (0.059)	0.362 (0.100)

Table S3 Comparisons of different approaches based on PC-stable for instant causalities in multi-trace simulations. The averages of 500 multi-trace simulations are shown with standard deviation values in brackets. The significantly best (t-test) results are marked in red.

N(0,0.01) (low-noise)	CITL	Scribe (top)	Scribe (AUROC)	Scribe (AUPR)
**Discovered edges**	24	24	0.899	0.854
**Real edges**	13	13		
**Correct directions**	13	13		
**N(0,1) (high-deviation)**	**CITL**	**Scribe (top)**	**Scribe (AUROC)**	**Scribe (AUPR)**
**Discovered edges**	20	20	0.957	0.902
**Real edges**	14	13		
**Correct directions**	13	13		
**N(5,0.01) (high-mean)**	**CITL**	**Scribe (top)**	**Scribe (AUROC)**	**Scribe (AUPR)**
**Discovered edges**	28	28	0.755	0.612
**Real edges**	9	8		
**Correct directions**	2	6		

Table S4 Comparisons between CITL and Scribe under the simulation setting of Qiu et al. with three different noise levels.

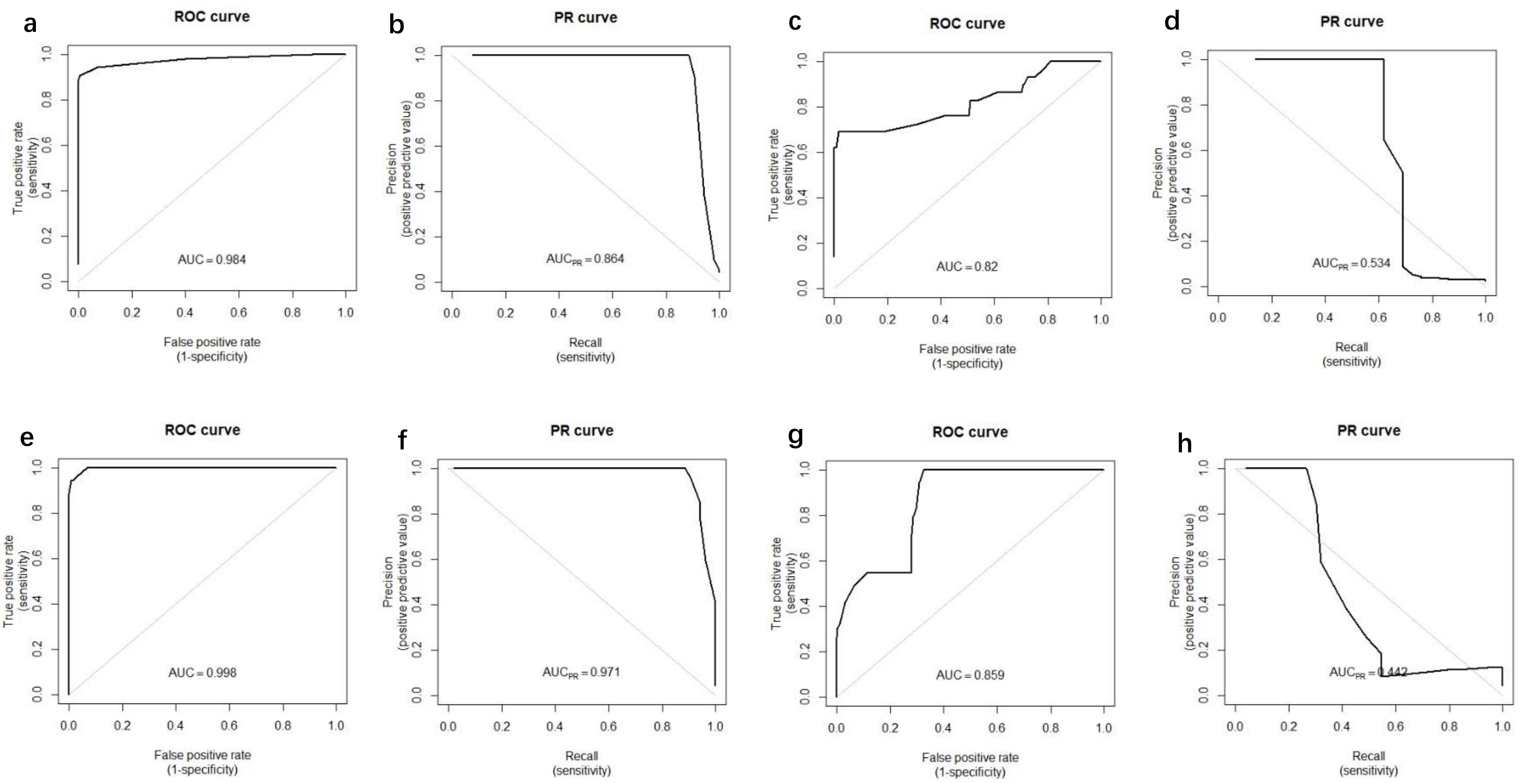

Figure S1. ROC and PR curves of Scribe and Approach 0 in single- and multi-trace simulations. (**a, b)**, the curves of Scribe in the single-trace simulation; (**c, d)**, the curves of Scribe in the multi-trace simulation; (**e, f)**, the curves of Approach 0 in the single-trace simulation; (**g, h)**, the curves of Approach 0 in the multi-trace simulation.

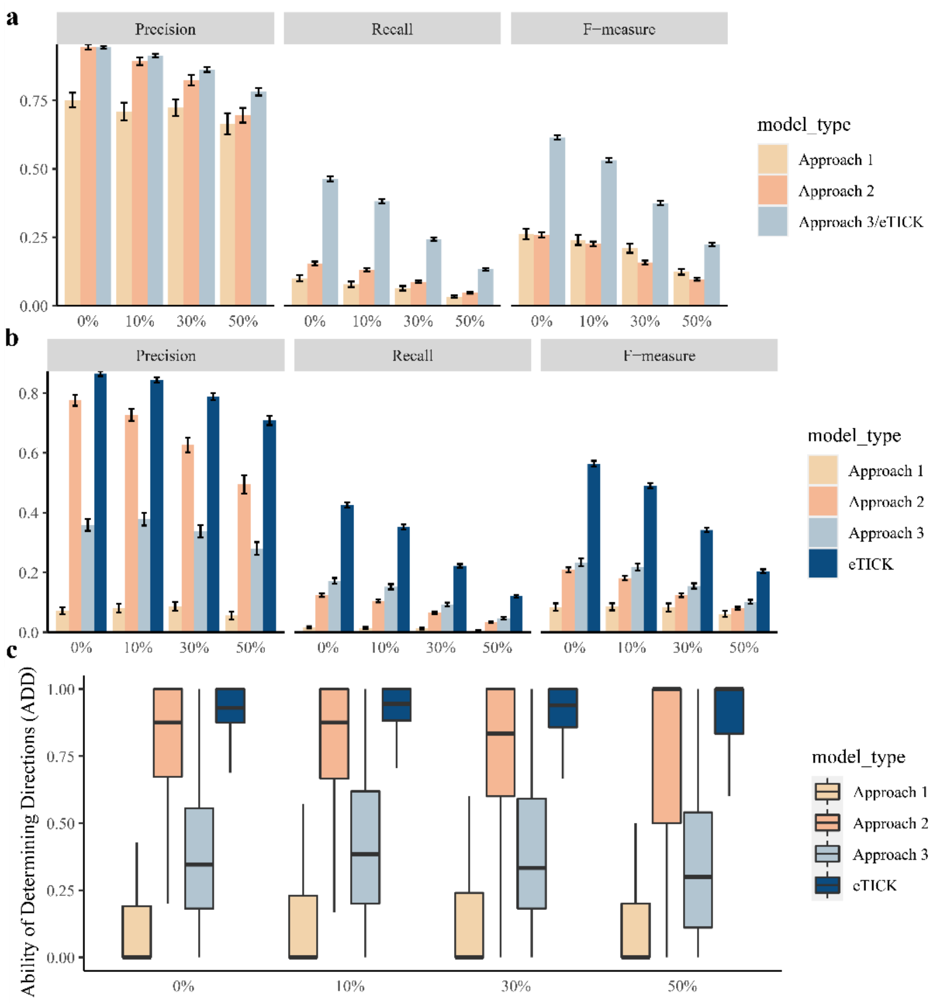

Figure S2. Results of multi-trace simulation with latent variables. a, the performance of discovering edges; **b**, the performance of determining directions; **c**, ability of determining directions.

As described in Materials and Methods, there are two sources of variations: the variation from initializing the previous expression levels ($I_{variance}$) and that from transferring time-lagged causality ($C_{variance}$). In the manuscript, $I_{variance}$ and $C_{variance}$ are set at 0.2 and 1, respectively.

$$\ln\left(X_i^{pre}\right) \sim N\left(0, {I_{variance}}^2\right)$$

$$e^{cur},\ e^{sub} \sim \mathrm{N}\left(0, {C_{variance}}^2\right)$$

We compared the performance of different approaches in simulations for different combinations of the two variances. Note that the edges recovered by Approach 3 is the same as those of CITL.

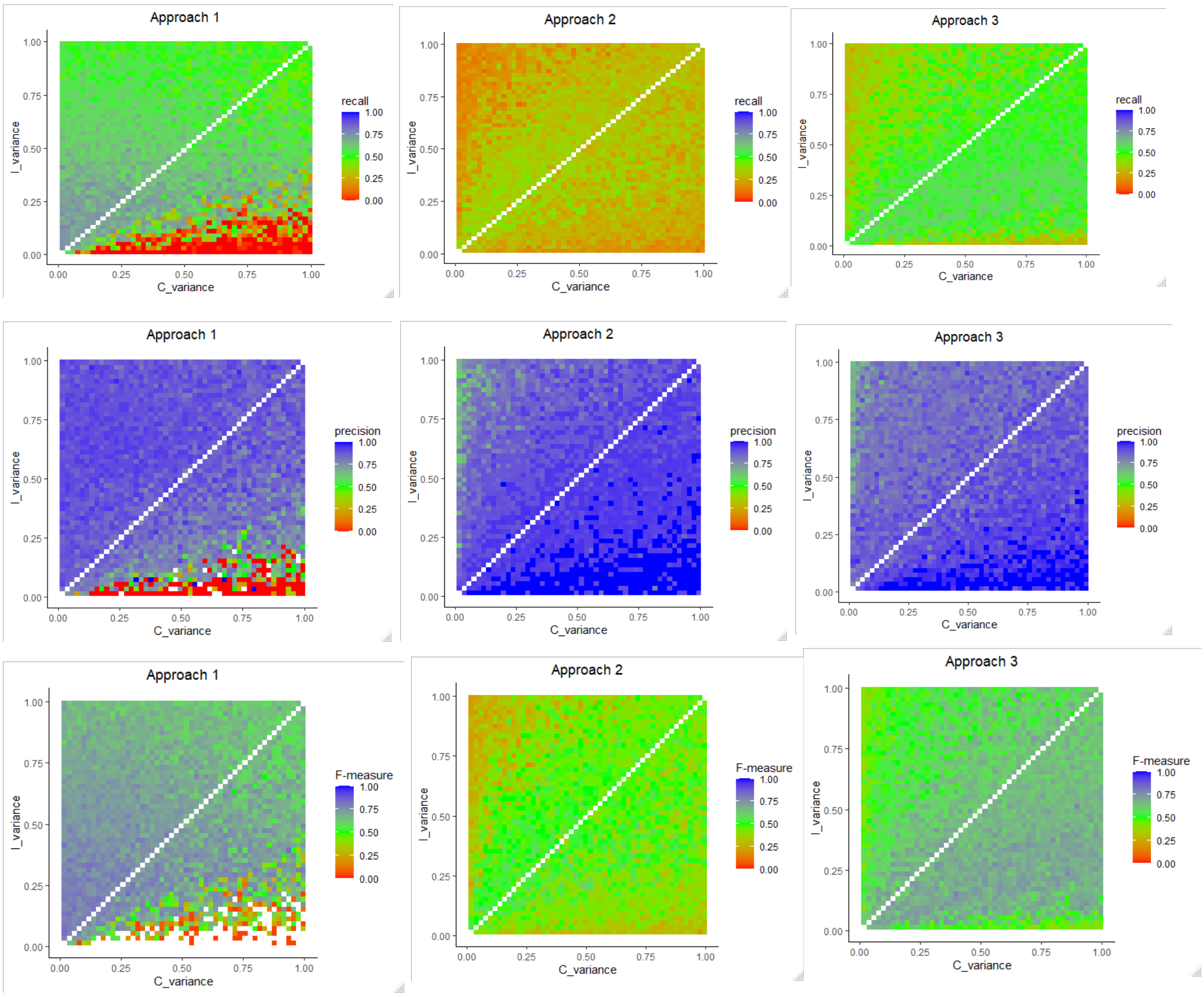

Figure S3. The performance of discovering edges in single-trace simulations.

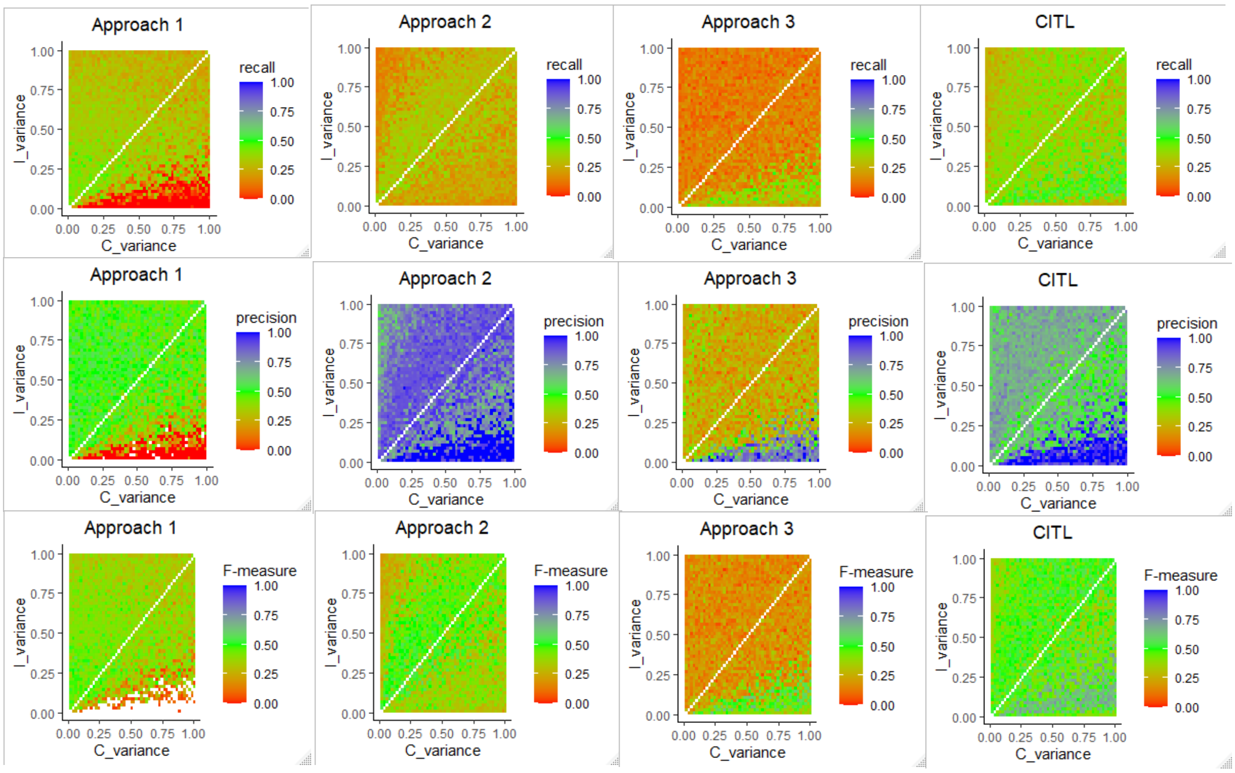

Figure S4. The performance of determing directions in single-trace simulations.

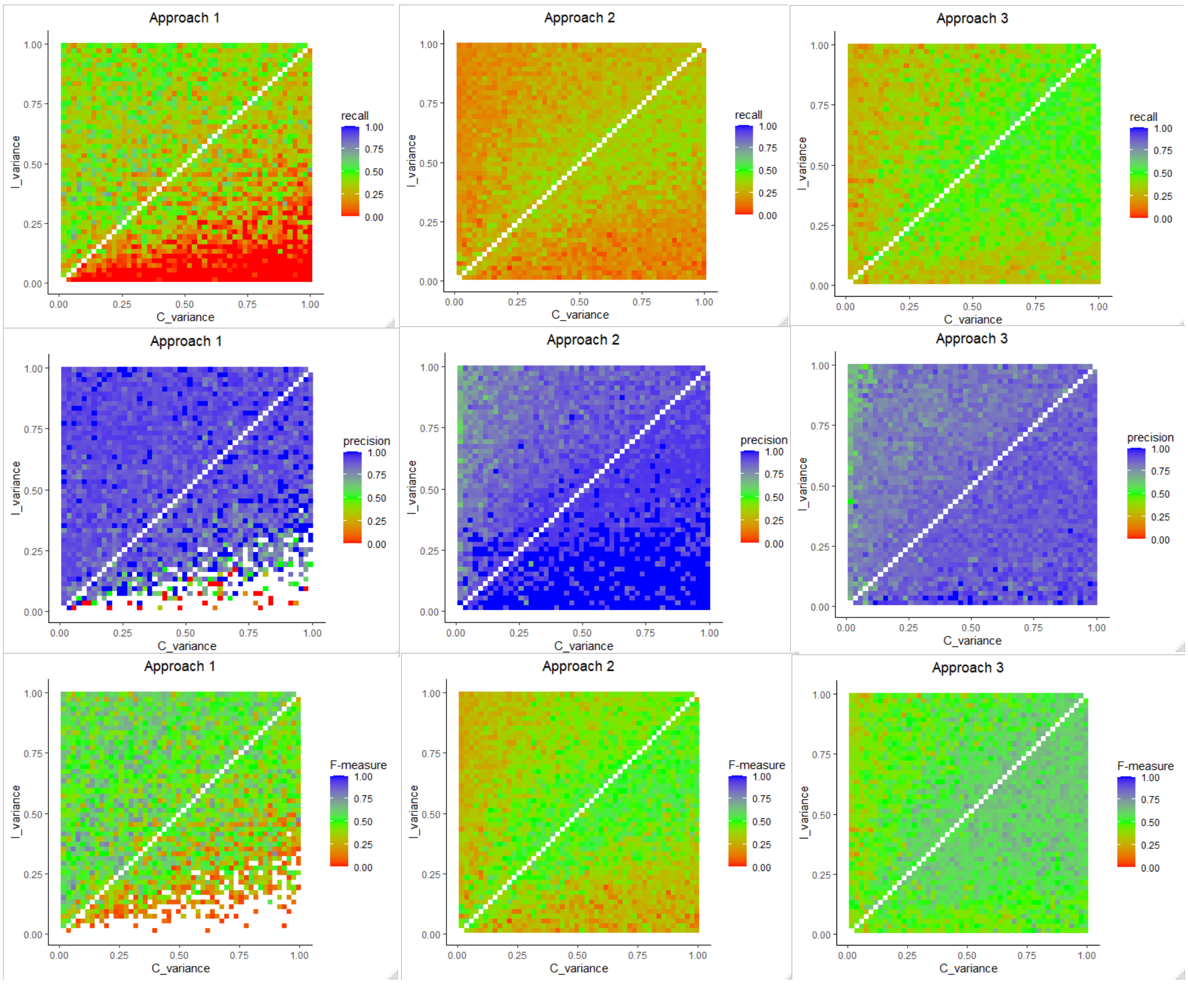

Figure S5. The performance of discovering edges in multi-trace simulations.

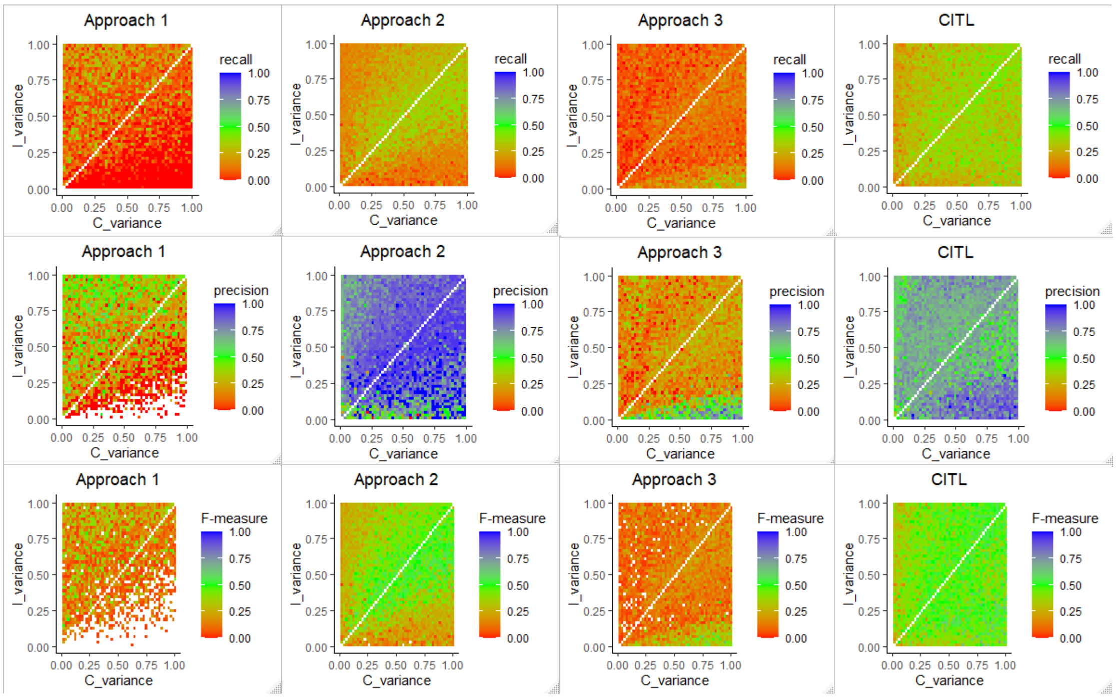

Figure S6. The performance of determing directions in multi-trace simulations.

We applied Approach 0 to data set 1. The cutoff of the absolute Pearson's correlation coefficient was set to 0.85, and the indegree and outdegree were calculated. Given a specific gene, the indegree is the number of its causes while the outdegree is the number of its effects. Eight thousand four hundred eighty-six causal pairs were assigned with only seven bidirectional pairs. There were 342 genes with their indegrees larger than 0 and 1,435 genes with their outdegrees larger than 0. The distributions of indegrees and outdegrees are presented in Figure S7a. There were more genes in which the indegree was 10-fold higher than their outdegree (the bottom-right n red dots in Figure S7b). The unbalance can be interpreted as that many other genes highly regulate these genes due to their essential roles. Similar results were obtained for data set 2 (Figure S7c, S7d).

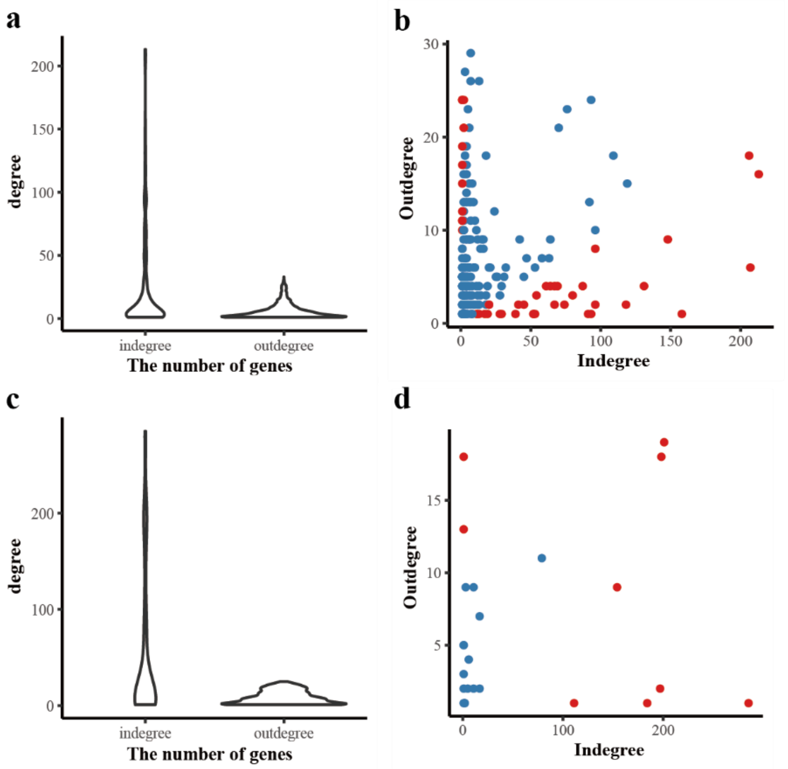

Figure S7. The distributions of the indegrees and outdegrees inferred from Approach 0 in data set 1 (a, b) and data set 2 (c,d).

We performed gene ontology enrichment analysis to investigate the function of the causal pairs discovered by Approach 0 with data set 1, using *clusterProfile* package (Yu et al.). The top three enriched molecular functions were (adjusted *p*-value, p_{adjust} < 1E-10) ion gated channel activity, gated channel activity, and substrate-specific channel activity. Because the experiment was performed on developing mouse hippocampus cells, it is reasonable that most of the genes we found were related to nerve impulses. For the genes with indegree higher than 15 (n = 108), the top five enriched molecular functions were motor activity (p_{adjust} = 0.033), ATP-dependent microtubule

motor activity (p_{adjust} = 0.055), tubulin binding (p_{adjust} = 0.055), ATPase activity (p_{adjust} = 0.055), and guanyl-nucleotide exchange factor activity (p_{adjust} = 0.055). This shows that the functions of genes with high indegree are different from the other genes. Only one molecular function was significantly enriched (motor activity), thus showing the functions of these genes were distributed in multiple fields. If a gene is regulated by many others, it might be vital for cell survival, such as house-keeping genes. Important genes are likely to have different functions; this was in accordance with the dispersion of our enrichment results. On the other hand, genes with an outdegree of one (n = 343) were enriched with other molecular functions, including microtubule binding (p_{adjust} = 0. 003), catalytic activity, acting on DNA (p_{adjust} = 0. 003), tubulin binding (p_{adjust} = 0. 020) and single-stranded DNA binding (p_{adjust} = 0. 020). The second and fourth functions both involve DNA, suggesting that these one-effect causes identified by Approach 0 might participate in the regulation of gene expression.
